# The role of doulas in supporting perinatal mental health – a qualitative study

**DOI:** 10.3389/fpsyt.2024.1272513

**Published:** 2024-02-29

**Authors:** Joanne Quiray, Elizabeth Richards, Yesenia Navarro-Aguirre, Debra Glazer, Jamie Adachi, Emily Trujillo, Dila Perera, Elizabeth Perez Garcia, Amritha Bhat

**Affiliations:** ^1^ Department of Psychiatry and Behavioral Sciences, University of Washington, Seattle, WA, United States; ^2^ Open Arms Perinatal Services, Seattle, WA, United States

**Keywords:** perinatal mental health, maternal mental health, doula, screening, qualitative, needs assessment

## Abstract

**Objective:**

The perinatal period presents several opportunities to identify and treat perinatal mental health and substance use disorders by integrating into existing care pathways. We aimed to examine the role of birth doulas in supporting their clients’ perinatal mental health.

**Methods:**

Thematic content analysis of focus groups with doulas, and interviews with doula clients was used to characterize the doula-client relationship, investigate whether and how doulas provide mental health and substance use support, and identify barriers and recommendations for doulas to support their clients’ mental health. Participants were doula clients from communities underserved due to race, income, language and culture.

**Results:**

Doulas and clients reported positive relationships, supported by congruence in culture, language, and lived experiences. Doulas varied in their confidence in identifying perinatal mental health problems, though most agreed that doulas could support their clients’ mental health to different degrees. Barriers to engaging in perinatal mental health treatments included low perceived need and socio-economic burden.

**Conclusions:**

With adequate support and training, doulas can play an important role in supporting their client’s emotional well-being.

## Introduction

Perinatal (pregnancy and the first year postpartum) mental health (PMH) and substance use disorders (SUD) are the most common complications of pregnancy yet frequently go undetected and untreated, especially among historically underserved communities such as Black, Latina/o/x, Indigenous, and people of low income. Perinatal mental health conditions include depression, anxiety, obsessive compulsive disorder, post-traumatic stress disorder, bipolar disorder and psychosis. Black and Latina/o/x women are less likely to receive mental health screening (1), assessment (2, 3), or treatment (4–7) than White women. These inequities persist after adjusting for education and income (8) and have been exacerbated by the COVID-19 pandemic (9). Factors contributing to this treatment gap and inequities, include mental health workforce shortages (10), lack of culturally congruent providers (11) structural racism resulting in mistrust in the medical system (12), employment, income, transportation, insurance status and type, refugee status, and immigration status (13). Addressing subjective perceptions of need for mental health treatment is also important as low income mothers and pregnant people with psychological distress may be reluctant to engage with mental health services due to a belief that only severe mental health conditions warrant treatment (14). Social norms surrounding discussion and treatment of mental illness and stigma may vary by race and ethnicity (15), and underserved people can experience the “double stigma,” of mental illness and discrimination, contributing to lower rates of treatment seeking (16). Black and Latina/o/x women may be more likely than non-Hispanic White women to report perceived stigma about depression (17), or to believe that mental health treatment will be unhelpful (18). They may be less likely to find antidepressant medication (19) and counseling (20) acceptable. Mothers with income inequity have reported that mental health treatment would do nothing to address their psychological distress, which they viewed as due entirely to external stressors such as poverty (14).

Barriers to PMH treatment access can be addressed by integrating care into community-based settings, with the support of trusted individuals such as doulas. Birth doulas are individuals from the same community as their client, who are trained to provide psychosocial, emotional, and educational support during pregnancy, childbirth, and postpartum, and act as a liaison between their client and the health care system (21). They help people navigate systemic healthcare racism and socioeconomic barriers (22–24). Including doulas in prenatal care improves pregnancy and birth outcomes (25, 26) and patient satisfaction (27), and is a promising approach to address racism and inequities in maternal health.

Doulas can potentially play a greater role in the identification and initial support of clients with PMH and SUD as they interact frequently with their clients through the perinatal period and develop longitudinal, trusting relationships. Screening, education, and support offered by doulas may be more acceptable to clients, without the barriers of stigma and mistrust of the healthcare system. Studies describe the emotional support provided by doulas during labor and delivery (28), and in the postpartum period (29), and doula’s PMH training needs (30–33). Our aim in this qualitative analysis is to understand the role of doulas in perinatal mental health, from the perspective of both doulas and clients.

## Materials and methods

This qualitative study includes focus groups with birth doulas (referred to as doulas here) and individual interviews with clients. We obtained Institutional Review Board (IRB) approval and collaborated with Open Arms Perinatal Services (OAPS) to recruit study participants. OAPS is a non-profit organization in King County, WA that provides services to clients living within 200% of the federal poverty level. It supports over 500 pregnant people and babies each year, 90% of whom are from underserved communities (34). We used convenience sampling to identify doulas and their clients using a combination of active(potential participants were contacted by phone and informed about the study) and passive(distributing fliers through the internal listserv) recruitment strategies. All participants provided informed consent and received $30 per hour compensation.

### Conceptual model

Focus group and interview guides were developed in collaboration with OAPS, based on the potential role for non-specialty health workers to be involved in their clients’ PMH care (35). In doula care, the role of doulas as culturally congruent liaisons between health care providers and clients may offset tensions arising from systemic racism and race/culture discordance between provider and patient (21). However, this pathway does not address other barriers to care that are common among populations experiencing disparities, including low perceived need for care, low awareness of services, low perceived access to care, low perceived effectiveness of care, and stigma (13). Hence, our interview and focus group questions were designed to examine the role of doulas in addressing these barriers and specifically in educating, screening, and referring clients with PMH and SUD concerns.

### Data collection

To limit bias that might be seen in a single group, we planned to conduct focus groups with doulas until we reached saturation. The focus group interviews were conducted by a facilitator separate from the study team, and an observer (YN) who made field notes. Three focus groups lasting 60-90 minutes each, with one, two and six participants respectively, were conducted between June 2022 and July 2022 in a Zoom virtual meeting room. To reduce stigma and encourage self-disclosure we conducted individual interviews with doula clients between July 2022 and September 2022 until saturation was reached. Individual interviews in English and Spanish conducted by JQ and YN lasted between 30-60 minutes. Transcriptions were created from the recorded focus groups and interviews, and Spanish language interviews were translated.

### Data analysis

Coding and analysis were performed by JQ, ER, YN, DG and AB. JQ is a research coordinator and identifies as a queer, second-generation Filipino American. ER is a Black female psychiatry resident physician, YN is first-generation Latina research coordinator. DG is a White female health services research coordinator. AB is a female perinatal psychiatrist and health services researcher who is a first-generation Asian immigrant. Using transcriptions as data, thematic content analysis combined with rapid team-based qualitative analysis were carried out using a process of progressive data reduction to narrow the focus and generate more specific codes from general codes (36, 37). Themes derived from interview questions were transferred to a doula and client summary template. The research team assessed and edited summary templates for agreement through coding of one identical doula and client transcript, then independently coded different transcripts. Themes and codes were revised through an iterative process in team meetings until consensus was reached. Results were presented to doula participants for feedback at a team meeting.

## Results

Doula and client participant demographics are summarized in **Table 1**. All participating doulas owned an independent practice and/or were contracted with an organization. In total, 28 doulas were contacted, and nine doulas consented to participate. A total of 44 doula clients were contacted (18 of whom were Spanish speaking) and 10 participants consented to participate. Of the ten participants included, five were interviewed in Spanish. All clients identified as female and received care from birth doulas who primarily serve during pregnancy and birth with a few postpartum visits. Eight out of nine clients had a doula for the first time; one client had the same doula for three consecutive pregnancies. All clients were postpartum at the time of the interview.

**Table 1a:** Birth doula demographics.

Focus Group	Years in practice	Race
A	9	Another Hispanic, Latino/a, or Spanish origin
B	3	Black
B	3	Another Hispanic, Latino/a, or Spanish origin
C	1	White
C	6	Black
C	6	Mexican
C	10	White
C	1	Black
C	7	Black

**Table:** Table 1b Client demographics.

Participant	Interview language	Race	MH history	Pregnancy stage	Prenatal care receipt
1	English	White	Depression symptoms	7 weeks postpartum	OB/GYN
2	English	Dominican and Black	None	16 weeks postpartum	Midwife
3	English	Black	Alcohol use before pregnancy, current depression symptoms	24 weeks postpartum	OB/GYN and Midwife
4	English	Black	Depression symptoms and stress	20 weeks postpartum	OB/GYN
5	Spanish	Mexican	None	12 weeks postpartum	OB/GYN
6	Spanish	Hispanic, Mexican	None	20 weeks postpartum	Primary care
7	Spanish	Latina, Peruvian	None	24 weeks postpartum	OB/GYN
8	Spanish	Latina, Mexican	None	12 weeks postpartum	Midwife
9	Spanish	Mexican	None	20 weeks postpartum	Midwife

### Themes

We examined the role of doulas in perinatal mental health through four themes, with a total of ten codes that emerged from doula and client narratives (**Table 2**).

1. Doula – client relationship (n=3): nature of relationship, quality of relationship, and congruence
2. Social support and PMH and SUD (n=1): PMH and SUD stigma in communities
3. Doula’s response to client’s PMH and SUD (n=4): education, approach to identification of PMH and SUD, variability in capacity to manage clients’ PMH and SUD problems, and referrals to services and treatments
4. Barriers to PMH and SUD treatment (n=2). Low perceived need for care and low perceived access to care and client unable to access PMH care to due socio-economic burden

**Table 2:** Qualitative findings from focus groups with doulas and interviews with clients.

Theme	Code	Quote from doula	Quote from doula client
Doula – client relationship	Nature of relationship	“…it’s important from my perspective to show my clients to navigate in the system, for them to know what to expect there. My advocacy is not to speak for them but to say, “You can do this. You have the power to do this, and remember that all the time…” (A) “And that was two times where Mom had postpartum depression … and I was able to help her to see what she was going through and to recognize that was not just in her head … And it had a name, and it needed medication, and it needed support. And she got it.” (C)	“I was going through some difficult emotional challenges and changes in my life during the pregnancy, and she was really there to be a sounding board and to help me prepare for what my life might look like in different situations and just to have compassion and empathy … I had to have a medical appointment… – and she was able to help attend that appointment with me and help me get the kids in the stroller and do things that were physically challenging for me because I was just a few days’ post C-section.” (Participant 1) “She was there when I had my child and was an advocate” (Participant 4) “…she also does the translator service. So, there were things that my husband didn’t understand like hospital things, health; but she helped, and, in fact, the doctors said that: “Oh, that’s very good,” and she said, “We have two for one”, because she helped as a doula and helped as a translator.” (Participant 5)
	Quality of relationship	“….you also become her counselor, her confidante, her friend, her sister, and sometimes her aunt” (C)	“I thought I was going to feel a little uncomfortable because … it was only by zoom. But no, she is nice, she was very supportive and helped me a lot” (Participant 8) “It was very friendly. Like if she was like an aunt…. who was taking care of me.” (Participant 9)
	Congruence	“I feel I really can’t understand my clients unless they are Latina because I am Latina. I am an immigrant, and I can know how all my community is going to be similar” (A) “A lot of my clients do identify as Black. And they want to have someone in their space that looks like them and has that in common” (B) “I decided to become a doula because I have been working in the deaf community for the last eight years. And as I became friends with more and more deaf people, I started hearing a lot of really traumatic birth stories about lack of access in their native language in birth work” (C)	“she’s also Hispanic, we didn’t know which country, but we know she’s Hispanic. And no, the truth is that she helped a lot, she’s an older person, which that gave me that trust, for me it was as if my mother was with me…” (Participant 5) “The fact that she spoke Spanish, obviously, and that she is of my same race, you could say, because she is Argentinian. So, it wasn’t a person from here who speaks Spanish with that accent … So, everything flowed from the moment we had the Zoom meeting, before we met in person, and the moment when she was already there was as if we knew each other for a while. I think it was because of that why she understood perfectly what I was saying.” (Participant 7) “My doula was black. I’m white, but my husband and all my children are black, and so it might not necessarily be that she connected with me culturally, but I felt like I had a connection with her because she respected my family structure. I knew she respected the interracial marriage. I know that she respected black people and the situations that they deal with that white people don’t have to deal with and the situations that we might encounter being an interracial couple or having black children…” (Participant 1)
Social support and PMH/SUD	PMH and SUD stigma in communities	“You don’t want to express it to anybody … those symptoms aren’t really discussed because of the stigma around them” “they didn’t want to accept that they have a problem…, for Hispanics, seeing a counselor, not a chance, “Oh, I am not crazy.”… and that’s why they don’t seek that type of help and because they don’t have enough information, either” There are “stigmas around Black mental health…. it’s a new concept to seek outside support and being honest about what you’re experiencing … There’s just been a lot of secrecy when it comes to mental health stigmas” (B) “…with my community [Latino/a/x], it’s really a taboo talking about mental health, and this could be really offensive for some people. So, we have to be really care about the way that we are asking or presenting something.”	“…the one I’ve talked about those aspects with is my mom. She told me that if I felt some symptoms, to talk to my doctors. Or, in this case, also with my husband so that he could help me make some kind of decision in case something like that would happen to me or similar aspects.” (Participant 6) “My family was afraid that I would get postpartum depression because … they heard me more than anything talk when I was pregnant: ‘Please let it be over’…they told me: ‘When you give birth you have to have willpower, you might get this, you might get that.’ And I was like, “Well, I hope I don’t get it because it’s not something I can control.” (Participant 7)
Doula’s response to PMH/SUD	Education	“…everything education-based … after they’ve given birth, I let them know those first three days there’s a big hormone influx, so you’re going to feel a lot of different feelings. And then, in about two weeks, I’ll check in with you again and see how you’re feeling … if you’re feeling these intrusive thoughts about maybe wanting to cause harm to your baby … just letting them know what those symptoms…” (B)	
	Approach to identification of PMH/SUD	“I really like to give them a questionnaire to complete. On the questionnaire she put more things than she told me in person.” (A) “I would say that having a questionnaire or paperwork for my clients is not appealing. I try to give it as a way of empowerment and for them to utilize … but I would never feel comfortable saying, “I want you to complete this PHQ9 as a way for me to gauge how you’re doing.” (C) “You get to know this person. And when something is not right, you would know something is not right … as for substance abuse, I did have a client… – she confided me – that she was using I got her into rehab … she got the help that she needed.” (C)	
	Variability in capacity to manage clients’ PMH and SUD problems	Regarding substance use, “that was the very first time that I encountered that problem”. “I had to turn a client away because her level of anxiety and depression was too much for me and I just didn’t know how to handle it” (B) “So, I kind of usually avoid when I see clients with substance abuse, because I was scared that I was not able to help my clients since I did not have any experience of this field” (C) “It’s really hard for me because I don’t feel I have the tools to help … I don’t have a really good response in the middle of the night” (A) “Everybody [doulas] turned her down because nobody was prepared to deal with that amount of stress that she was going through.” (B) “I did have a client … [confide in me] that she was using … So, I got her into rehab … and continue to seek counseling and continue to be monitored … I actually became kind of a big sister to that human being … I make sure that every step, I was there to support and to help her.” (C) “just life experience … [knowing] people who are dealing with substance issues … it’s just learning who they are, learning about their story, creating a safe space for them to feel open enough to say what’s going on…” (B)	“…we also touch on mental health issues and things like that; she would ask me how I was feeling, and sometimes she would tell me that it was a bit normal to feel overwhelmed when the baby and the sibling were first born. She also shared her experiences and I believe that the fact that she also opens up with you generates more confidence to tell her my things as well.” (Participant 8)
	Referrals to services and treatment	“…I really like to connect them with some particular services. I like to offer them this Perinatal Support Washington…” (C) “I want to help you. In order to help you we need to go through my organization to look for the best resources for you,” (A)	“…she reached out and she spent time trying to research things and find providers and call places and give me resources.” (Participant 1)
Barriers to PMH and SUD treatment.	Low perceived need for care and low perceived access to care	“In their opinions, they either are not getting the help, or they don’t know that there is help … I think a lot of my clients didn’t know what the symptoms are. They think it’s just a part of the experience for the first-time moms, or even the second-time moms because, the nature of mood disorders looks different, and it varies per pregnancy” (B)	“I didn’t actively look for a mental health therapist or anything because I felt like I needed money, like, my own money … I think I had already put it in my mind that I wasn’t gonna be able to get it” (Participant 3)
	Client unable to access PMH care to due socio-economic burden	“I see that mental health can come up as something that is struggling at any point during pregnancy or postpartum, but it has to be maybe lower on the priority list than it … could be for someone who has more of their basic needs met” (C)	“…there’s no psychiatrists around here that take it, so that has been a big problem why at times when I definitely felt like I needed more mental health care or was really struggling, I just couldn’t get it.” (Participant 1)

PMH, Perinatal Mental Health; SUD, Substance Use Disorder.

### Doula – client relationship

Both doulas and clients reported positive relationships, supported by congruence in culture, language, and lived experiences. In addition to labor and delivery support, doulas provided advocacy, translation services, decision making support and logistical support.

Participant 1 had the same doula from two previous pregnancies and appreciated that rapport during virtual care visits with their most recent pregnancy due to COVID-19 restrictions.

*“…[it’s]hard to make that bond, especially in a pandemic, and [to] have that connection with someone new. So, being able to have that same support person I think was really, really, really helpful for me.”*

Cultural and linguistic congruence between Spanish-speaking doulas and clients supported strong relationships, especially for some whose immediate families lived outside of the United States. Congruence in lived experiences was also important. A client in an interracial marriage felt respected and understood by her doula. A doula who had previously worked in the deaf community utilized their experience to provide more accessible services.

Only one client reported a negative relationship with two doulas due to inconsistent care from the first doula and disagreement during their birth with the second doula.

*“Her phone[was] always messed up … she couldn’t even come to the[birth] … so then I had to get the hospital doula, and my doula wasn’t as holistic … when I was talking about having a placenta birth, she kind of made a face … [I was going to]say a lotus birth and she didn’t understand what that was, and she was taken aback … she kept throwing little things out there to say, ‘You still want to do that?” *(Participant 2)

### Social support and PMH and SUD

Within clients’ communities, cultural norms and beliefs impacted perceptions of PMH and SUD. Several doulas also described the lack of discussion and treatment of PMH primarily in communities of color. Two Spanish-speaking clients stated their families believed PMH symptoms could be controlled through self-management.

*“I did recently tell my mother: “Oh, mom … I feel bad or I feel this”, she told me: “No, just sleep well, eat at your own time, be calm, don’t worry…” *(Participant 5)

Many clients stated that PMH and SUD were not explicitly discussed in their social networks, though they believe their families would support treatment and discourage use of substances. Two clients with self-reported PMH symptoms openly discussed their PMH within their communities more so during postpartum and were able to receive support from their families.

*“So, I had that at that time [in home country], but just having my spouse there … always cheering me on, and taking me out … and also removed the stress of caring for the baby because my mom was also like, ‘she can’t just stay with the baby’…” *(Participant 4)

Both doulas and clients agreed that it was important for clients to feel close and safe with the people they discuss PMH and SUD challenges with.

### Doula’s response to client’s PMH and SUD

Two of three clients with PMH symptoms reported that their doulas discussed PMH with them and provided resources. The third client’s doula provided emotional support but did not explicitly discuss MH. All but one of the clients who did not report symptoms would be willing to discuss their PMH with their doula if they were to experience it and would involve their doula in PMH care if given a choice. The one client who was hesitant stated it would be contingent on having a positive relationship with their doula.

One doula reported challenges with adding PMH support to her existing responsibilities *“Man, I’m trying to handle the induced labor and we were having trouble … And then, I have these other clients calling me off the hook. My phone is ringing off the hook.”*(C)

Some doulas preferred to use standardized questionnaires when evaluating PMH. Others used informal screening questions:

*“…are you getting enough rest? Are you sleeping well? Are you making sure you[are] letting your body heal?”*(C)

One doula described their hesitancy with using standardized questionaries because they felt that utilizing questionnaires would align them more with institutions rather than serving as an advocate for the client.

Some of this variability in the capacity to manage PMH and SUD symptoms amongst the doulas was attributed to lack of experience. Doulas who felt confident in the management of PMH and SUD reported that they had a strong rapport with their clients, received additional training, and/or had lived experiences with PMH and SUD. Upon recognition or identification of PMH or SUD, most of the doulas made referrals to available services and treatments. Both doulas and clients noted additional referral support from doulas such as assistance in making phone calls, check-ins about referral completion and encouragement to continue treatment. One doula who noted a specific interest in PMH started a support group for Black clients.

### Barriers to PMH and SUD treatment

Doulas reported barriers to supporting their clients’ PMH included their own lack of knowledge and comfort, and dearth of appropriate referral options. Doulas found that stigma and fear of consequences can prevent clients from accessing care and further barriers include insurance, scheduling difficulties, cultural congruence with provider and client, and appointment adherence. Doulas also observed a de-prioritization of PMH symptoms among clients struggling to meet basic needs.

Clients were open to having doulas attend their PMH treatment appointments to facilitate trust and communication as needed, particularly if they had a trusting relationship with the doula *“…if she were in a very intimate moment of my life, which is like giving birth, without her being my family I gave her that confidence and she gave me her support, I feel that she would also be part of the family and I could ask her to accompany me to that type of appointments.” *(Participant 6)

Unique factors were identified related to the fact that doulas were from the same community as their clients. One doula reported being cautious about broaching the topic of PMH with their clients because of the stigma associated with it. *“So … with my community, it’s really a taboo talking about mental health, and this could be really offensive for some people. So, we have to be really care(ful) about the way that we are asking or presenting something.”*(C)

## Discussion

In this qualitative analysis of the role of doulas in supporting their clients’ PMH, we focused on participants from underserved communities. with and without a history of mental health symptoms.

Doula – client relationships were largely positive. Doulas provided support that extended well beyond labor and delivery, from instrumental support (helping clients get to their medical appointments), to emotional support (being a “sounding board”, almost like a family member). Support in the postpartum period was especially appreciated and desired. Doulas extended their role to support clients facing linguistic barriers by helping with interpretation. Importantly, helping patients advocate for the healthcare they needed and wanted emerged as a key role for doulas, with an emphasis on the importance of remaining within scope of practice.

The relationship was viewed as especially supportive when there was congruence between the doula and client in one or more aspects – e.g., race, language, or lived experience of being a parent or having experienced racism. Interestingly, even with racial concordance, the doula’s supportive personality was considered more important in nurturing a strong relationship. Among our participants who identified as Hispanic or Latina/o/x, despite being of different nationalities, the ability to converse with their doula in Spanish was a source of comfort. Congruence in age was viewed as beneficial by some, but for some, having a doula older than them, almost like a mother figure, was more important. Although doulas reported best being able to support clients from their own community, some doulas reported needing to be cautious in their approach and mindful of the stigma of mental health within the community. This contrasted with some participants reporting that they discussed their mental health issues with the doula but not with their family because of stigma. These findings highlight the unique benefits and challenges of receiving community based mental health support.

Although most doulas had were willing to support their client’s PMH and SUD to different extents, some doulas avoided taking on clients with PMH and SUD due to feeling unprepared, highlighting the need to provide education and clear referral pathways so doulas can effectively build on their trusting relationship with clients to facilitate PMH and SUD treatment. Additionally, doulas explained the difficulties they experienced trying to manage the complexities of their clients’ PMH needs while attending to their usual duties of providing labor and delivery support. When involving lay or community health workers in the mental health treatment pathway it is important to address scope of practice and burden (38). Doulas provided education and anticipatory guidance about postpartum depression and intrusive thoughts, identifying PMH and SUD concerns, and provided resources. Notably, some doulas specifically preferred not to use structured questionnaires to screen for PMH symptoms. Several agencies have recommended screening for perinatal depression using structured questionnaires such as the Patient Health Questionnaire – 9 or Edinburgh Postnatal Depression Scale (39, 40), and screening using standardized validated questionnaires is common, feasible, and effective in community-based settings (41). However, our findings suggest that there may be a need to study additional approaches to support screening for PMH and SUD in the context of doula care.

Treatment access was limited by the lack of providers accepting their insurance. Doulas helped to the extent they could by following up on their clients’ PMH referrals. Barriers to PMH and SUD have been discussed extensively in the literature (42–44). In addition to commonly documented barriers, we found that perceived need for care and perceived access to care were low. Mental health was deprioritized due to socioeconomic burden, and participants felt that it was futile to look for PMH who accepted their insurance (usually Medicaid for our participants). Stigma regarding PMH was common and interfered with treatment access. In this context, the strong, congruent, familial relationship with their clients was conducive to identifying PMH and SUD, however doulas noted that they needed additional training and support to do this effectively and to liaison with the health care system.

Integrating mental health screening and intervention into community-based settings, within the context of maternal child health care is a promising approach to increasing detection and treatment of perinatal mental health conditions. This approach using task shared interventions delivered by community health workers or peers within existing maternal child health platforms is commonly used in several programs across the world (45).

Strengths of our study include the focus on underserved communities, obtaining the perspective of both doulas and clients, and the broad range of experiences reflected in our participant pool. Limitations include potential selection bias as participants who consented to the study may be more likely to have a positive attitude towards PMH and SUD, whereas the doulas who might not yet be confident with helping their clients with their mental health might not be well represented in the data. The willingness of doulas to support their clients’ PMH and SUD may depend on local availability of PMH resources and trainings, and findings from this single site study may not be applicable to all settings.

## Conclusions

We found that most doulas are already supporting their clients’ mental health in several different ways, addressing barriers to mental health treatment that may be unique to underserved populations. However, they report unmet identified perinatal mental health training needs and concerns about scope of practice and burden. Several states have legislation pending that would allow for Medicaid reimbursement of doula services, thus making doula services more accessible. This could be an inflection point for better coordination between healthcare and community-based systems, and an opportunity to decrease inequities in PMH and SUD treatment. With adequate support and training, doulas can play an important role in supporting their client’s emotional wellbeing.

## Data availability statement

The raw data supporting the conclusions of this article will be made available by the authors, without undue reservation.

## Ethics statement

The studies involving humans were approved by University of Washington Institutional Review Board. The studies were conducted in accordance with the local legislation and institutional requirements. The participants provided their verbal informed consent to participate in this study.

## Author contributions

JQ: Methodology, Writing – original draft, Writing – review & editing, Data curation, Formal analysis, Project administration. ER: Formal analysis, Writing – original draft, Writing – review & editing. YN-A: Formal analysis, Writing – review & editing, Validation. DG: Formal analysis, Writing – review & editing. JA: Writing – review & editing, Project administration, Resources, Supervision. ET: Resources, Writing – review & editing. DP: Resources, Writing – review & editing, Supervision. EG: Resources, Writing – review & editing. AB: Resources, Writing – review & editing, Conceptualization, Funding acquisition, Investigation, Methodology, Supervision, Writing – original draft.
