# Supplementary material for: The role of doulas in supporting perinatal mental health – a qualitative study
Source: Front Psychiatry. 2024 Feb 29;15:1272513. doi: 10.3389/fpsyt.2024.1272513 (PMC10937562; doi:10.3389/fpsyt.2024.1272513)
Supplement: Supplementary file 1 [file DataSheet_1.docx]

**CLIENT INTERVIEW GUIDE**

**Introduction, Voluntary disclaimer, Recording consent**

**- START THE RECORDING –**

1. How are you doing today?

**Intro Prompt: First, we’ll go over some general questions about your background.**

1. What race / ethnicity do you identify with?
   1. American Indian or Alaska Native, e.g., Puyallup, Duwamish, Yakama, Dine/Navajo, Blackfeet, Mayan, Aztec, Inupiat, Tlingit, etc.
   2. Another Hispanic, Latino/a. or Spanish origin (e.g. Guatemalan, Spaniard, Colombian, etc.)
   3. Asian Indian
   4. Black or African American, e.g., African American, Jamaican, Haitian, Nigerian, Somali, etc
   5. Chamorro
   6. Chinese
   7. Cuban
   8. Filipino
   9. Japanese
   10. Korean
   11. Mexican, Mexican American, or Chicano/a
   12. Native Hawaiian
   13. Other Asian, e.g., Pakistani, Cambodian, Hmong, etc.
   14. Other Pacific Islander, e.g., Tongan, Fijian, Marshallese, etc.
   15. Puerto Rican
   16. Samoan
   17. Some other race
   18. Vietnamese
   19. White, e.g., German, Irish, English, Italian, Lebanese, Egyptian, etc.
   20. I prefer not to answer
2. What gender do you identify with?
   1. Woman
   2. Man
   3. Non-binary
   4. Transgender
   5. Trans man/Transgender Man/FTM
   6. Trans woman/Transgender Woman/MTF
   7. Genderqueer
   8. Genderfluid
   9. Gender variant
   10. Questioning or unsure of your gender identity
   11. None of these describe me
   12. I prefer not to answer
3. What Sexual identity do you identify with?
   1. Straight/heterosexual
   2. Gay or Lesbian
   3. Bisexual
   4. Asexual
   5. Other
   6. I prefer not to answer
4. Are you pregnant or postpartum?
   1. If pregnant – how many weeks along?
   2. If postpartum – how many weeks postpartum?
   3. Have you been pregnant before?
   4. Other – if other, please specify
      1. @Interviewer: Pregnancy loss is an exclusion criteria. I'm so sorry to hear about that, thank you for letting me know. Unfortunately we cannot proceed with this interview as we cannot include people that have recently experienced pregnancy loss in this study. I hope that you have supports that are helping you through this time. Would you be interested in some [resources](https://uwnetid-my.sharepoint.com/:x:/r/personal/mcmh_uw_edu/_layouts/15/Doc.aspx?sourcedoc=%7BC404AA36-70B7-4059-837A-C5AEF9AA5CAD%7D&file=Pregnancy%20Loss%20%26%20Infant%20Death%20Resources.xlsx&action=default&mobileredirect=true)?
5. If you receive/d prenatal care, where do/did you receive it?
   1. Obstetrician
   2. Primary care physician
   3. Midwife
   4. Other

**Intro prompt: Next we’ll talk about your relationship with your doula.**

1. Why did you decide to work with a doula?
   1. How did you get connected to your doula? (referred by OB / PCP, self referral?)
   2. What has been your experience working with your doula?
   3. How would you describe your relationship with your doula?
   4. Are there similarities between you and your doula that impacted your relationship such as race, age, life experiences?
      1. Are there differences that impacted your relationship?
   5. Is there anything that you would have changed about working with your doula?

**Intro prompt: Now we’ll discuss your experiences during and/or after pregnancy.**

1. What do you know about mental health during pregnancy or postpartum?

If participant asks for examples: like depression and anxiety / like cannabis and methamphetamines

- 1. What do you know about substance use during pregnancy and postpartum?
     1. If participant asks for examples: like cannabis and methamphetamines
  2. Had you thought about it much?
  3. Maybe your family or friends talk about it?
  4. What treatments are you aware of that are used for depression and anxiety during pregnancy and postpartum?

1. What are your family’s thoughts about mental health and/or substance use during pregnancy or postpartum?
2. Have you ever been impacted by mental health conditions and/or substance use ?

If participant asks for examples: like depression and anxiety / like cannabis and methamphetamines

- 1. (if yes) have you experienced any of these conditions personally?
  2. (if yes) How was your experience with the mental health system?
  3. How about during pregnancy or after birth time period?
  4. (if no) do you know of anyone that has experienced mental health and/or substance use concerns in the pregnant and postpartum period?

1. If you had questions about your mental health and substance use during pregnancy or postpartum, did you talk to anyone about it?
   1. (if yes) Who did you talk to? Could you please tell me more about your discussion with them?
   2. (if no) Could you please tell me why not?
2. Were you aware of resources such as meeting with a psychiatrist, therapist, counselor, peer mental health support groups available to you if you needed mental health and/or substance use care?
   1. (if yes) What resources and services were you aware of?
   2. (if yes) did you use any of these resources and services?
3. Did you discuss your mental health and/or substance use with your doula?
   1. (if yes) Could you please tell me more about your discussion with them?
   2. (if yes) What was the result of the discussions, for example referral to a mental health clinician or prenatal provider for treatment?
      1. (if yes) did you follow up on the referral?
   3. (if no) Could you please tell me why not?
4. What kinds of discussions with your doula were most helpful?
   1. Informational (e.g. pregnancy symptoms, infant information, mental health information, symptoms of depression, side effects of medication); Supportive (e.g. emotional support)?
5. A lot of people experience barriers to accessing care especially with the mental health system. Did you experience any barriers (like transportation, cost, etc.) to accessing pregnant and postpartum mental health and substance use care?
   1. Was there anything done to support you in navigating those barriers?
   2. Is there anything that could have been done to help you get the care you needed?
   3. If given a choice, would you use your doula as a mental health and substance use support person for example attending therapy appointments with you?

**Closing (5 minutes)**

Thank you for coming and sharing your thoughts. That completes the interview. Do you have any final thoughts that you would like to share before I end the recording?

Thank you again for participating in this interview. I will stop the recording now.

- **STOP RECORDING**
