# Supplementary material for: The role of doulas in supporting perinatal mental health – a qualitative study
Source: Front Psychiatry. 2024 Feb 29;15:1272513. doi: 10.3389/fpsyt.2024.1272513 (PMC10937562; doi:10.3389/fpsyt.2024.1272513)
Supplement: Supplementary file 2 [file DataSheet_2.docx]

**FOCUS GROUP GUIDE**

**Introduction, Voluntary disclaimer, Recording consent**

**- START THE RECORDING –**

| **Theme** | **Question** | **Probe(s)** |
| --- | --- | --- |
| Intro Icebreaker | Q1: Please take a couple minutes to say why you decided to become a doula and where you received your doula training. Where do you take clients? |  |
| Characterize relationship with clients | Q2: Doula clients might find different aspects important in a provider such as age, life experience, culture, religion, etc. To your knowledge, did any of these or other aspects impact your relationship with your client? |  |
| Understanding of perinatal mental health and substance use disorders | Q3: What experience do you have caring for clients with mental health concerns? How about substance use concerns? | - Did you feel adequately prepared / trained / supported? |
| MH & SUD stigma | Q4: Is there stigma surrounding your client’s mental health and substance use conditions? | - In referring to perinatal mental health/ substance use disorder is there other language that would be less stigmatizing to your clients? |
| Awareness building | Q5: Do you help your clients think about their emotional wellbeing or mental health in the perinatal period? | - If yes, how? |
| Screening / treatment | Q6: Do you detect mental health and substance use problems in your clients? | - If yes, how? - If yes, are validated screening tools such as EPDS or PHQ9 or GAD7 used? - If yes, what are your next steps typically? - If yes, do you provide support, education, advice, or treatment on this topic? |
| Referral | Q7: Have you ever referred your client to specialty mental health and/or substance use care? | - If yes, how did that go? |
| Training | Q8: Have you received training in perinatal mental health and substance use disorders? | - What training did you receive? - Did anyone have a different type of training? - Was this part of your doula certification training or separate? |
| Training | Q9: Would you benefit from training in perinatal mental health screening, referral and treatment? | - If yes, what topics would you like to learn more about? - What format? (length, days, times, webinar, workshop) |
| Doula’s role in care pathway | **@Yesenia– screen share show visual**  The process of addressing perinatal mental health and substance use begins with screening and identification, and goes on to assessment followed by referral, treatment initiation, and adequate treatment. Take a minute to take a look at this visual which illustrates the care pathway.  Do you think doulas have a role in supporting their clients through this pathway? Where and how in the care pathway do you think you or other doulas could best support clients? | - Do you educate your clients about mental health and substance use disorders? - How do you / would you collaborate with medical prenatal providers (e.g., obstetrics, family medicine, midwives) in the mental health and substance use care of your clients? - How well connected do you feel with your client’s prenatal providers? Do you ever discuss the care of your clients with their prenatal providers? - Would you consider providing brief psychological support to those with milder symptoms while they are waiting to see a mental health provide - **If your client is hesitating to meet with the mental health provider would you, with their permission, join the appointment?** - Would you talk to a psychiatrist or other mental health provider to discuss the care of your client? - Is making a referral to psychiatry within your scope of practice? |
| Barriers to care | Q11: From your perspective, what do you think are common barriers to accessing perinatal mental health and substance use care? | - What strategies would be helpful to address these barriers? - What point(s) in the care pathways do people get stuck? |

**Closing (5 minutes)**

Thank you for coming and sharing your thoughts! That completes the focus group. Does anyone have any final thoughts that you would like to share before I end the recording?

Thank you again for participating in this interview. I will stop the recording now.

- **STOP RECORDING –**
